# [Retracted] Matrix metalloproteinase-1 promotes prostate tumor growth and metastasis

**DOI:** 10.3892/ijo.2026.5850

**Published:** 2026-01-19

**Authors:** Sai Murali Krishna Pulukuri, Jasti S. Rao

Int J Oncol 32: 757-765, 2008; DOI: 10.3892/ijo.32.4.757

Following the publication of the above paper, it was drawn to the Editor's attention by a concerned reader that certain of the lung images shown in Fig. 2b on p. 760 were strikingly similar to data in an article published by the same research group in the journal *Cancer Research*, which, in the meantime, has been retracted. Owing to the fact that the contentious data in the above article had already been published prior to its submission to *International Journal of Oncology*, the Editor has decided that this paper should be retracted from the Journal. The authors were asked for an explanation to account for these concerns, but the Editorial Office did not receive a reply. The Editor apologizes to the readership for any inconvenience caused.

